# FacialNet: facial emotion recognition for mental health analysis using UNet segmentation with transfer learning model

**DOI:** 10.3389/fncom.2024.1485121

**Published:** 2024-12-11

**Authors:** In-seop Na, Asma Aldrees, Abeer Hakeem, Linda Mohaisen, Muhammad Umer, Dina Abdulaziz AlHammadi, Shtwai Alsubai, Nisreen Innab, Imran Ashraf

**Affiliations:** ^1^Division of Culture Contents, Chonnam National University, Yeosu, Republic of Korea; ^2^Department of Informatics and Computer Systems, College of Computer Science, King Khalid University, Abha, Saudi Arabia; ^3^Department of Information Technology, Faculty of Computing and Information Technology, King Abdulaziz University, Jeddah, Saudi Arabia; ^4^Department of Computer Science & Information Technology, The Islamia University of Bahawalpur, Bahawalpur, Pakistan; ^5^Department of Information Systems, College of Computer and Information Sciences, Princess Nourah bint Abdulrahman University, Riyadh, Saudi Arabia; ^6^Department of Computer Science, College of Computer Engineering and Sciences, Prince Sattam Bin Abdulaziz University, Al-Kharj, Saudi Arabia; ^7^Department of Computer Science and Information Systems, College of Applied Sciences, AlMaarefa University, Diriyah, Saudi Arabia; ^8^Department of Information and Communication Engineering, Yeungnam University, Gyeongsan, Republic of Korea

**Keywords:** facial emotion recognition, UNET, EfficientNet, transfer learning, image processing

## Abstract

Facial emotion recognition (FER) can serve as a valuable tool for assessing emotional states, which are often linked to mental health. However, mental health encompasses a broad range of factors that go beyond facial expressions. While FER provides insights into certain aspects of emotional well-being, it can be used in conjunction with other assessments to form a more comprehensive understanding of an individual's mental health. This research work proposes a framework for human FER using UNet image segmentation and transfer learning with the EfficientNetB4 model (called FacialNet). The proposed model demonstrates promising results, achieving an accuracy of 90% for six emotion classes (happy, sad, fear, pain, anger, and disgust) and 96.39% for binary classification (happy and sad). The significance of FacialNet is judged by extensive experiments conducted against various machine learning and deep learning models, as well as state-of-the-art previous research works in FER. The significance of FacialNet is further validated using a cross-validation technique, ensuring reliable performance across different data splits. The findings highlight the effectiveness of leveraging UNet image segmentation and EfficientNetB4 transfer learning for accurate and efficient human facial emotion recognition, offering promising avenues for real-world applications in emotion-aware systems and effective computing platforms. Experimental findings reveal that the proposed approach performs substantially better than existing works with an improved accuracy of 96.39% compared to existing 94.26%.

## 1 Introduction

Human facial expressions, primarily manifested through facial features, hold considerable emotional significance (Huang et al., 2019). People instinctively understand these expressions as they represent an individual's emotions and demeanor during interactions. With technological progress, there's an increasing interest in equipping machines with cognitive skills, leading to research and debate in areas like human-computer interaction and computer vision. A particular focus is on emotion recognition via facial expressions, with applications in human-computer collaborative systems, responsive animation, and human-robot interaction (Oguine et al., 2022). The challenge of identifying and classifying human emotions has been a topic of interest in psychology, anthropology, and computer science. Some researchers propose universal emotional categories, while others emphasize the cultural impact on emotional perception and expression. Cross-cultural studies reveal both similarities and differences in emotional categorization (Lindquist et al., 2022).

Recognizing human facial emotions (FER) is crucial for digital applications, human-computer interfaces, and behavioral sciences (Gupta and Jain, 2021). Understanding the movements of facial muscles and their link to emotions is essential for creating effective classification algorithms. Classifying facial emotions involves feature extraction (Song et al., 2024) and classification methods (Zhu, 2023). Despite advancements, accurately identifying facial expression subtleties remains challenging, particularly in online settings (Zhang et al., 2020). Using image classification to detect and categorize emotions is an intriguing frontier in emotion recognition. Deep learning models, specifically convolutional neural networks (CNNs), stand out for image classification and can reliably classify emotions in images when trained on datasets of facial expressions (Canal et al., 2022).

Human facial emotion recognition has several medical applications. For example, Vignesh et al. (2023) uses a novel CNN-based model for emotion recognition for psychological profiling. The model incorporates U-Net segmentation layers within VGG layers to extract critical features leading to better performance compared to existing approaches on the FER-2013 dataset. Similarly, other studies also report the use of CNN for enhanced accuracy in facial emotion recognition (Sarvakar et al., 2023; Huang et al., 2023). The study (Sarvakar et al., 2023) introduces a neural networks convolutionary (FERC) approach based on the CNN model. An expressional vector is leveraged in the proposed approach to identify five facial emotions. Contrary to the single-level approach used in traditional CNN, FERC follows a two-level approach.

Along the same course, the authors (Huang et al., 2023) utilize a transfer learning approach where CNN and residual neural network for facial emotion recognition. Features are extracted using the residual network which is later used with the CNN model. The authors found important features to provide better performance. Features around the nose and mouth are reported to be critical features to obtain enhanced accuracy. Results report an 83.37% accuracy with the AffectNet model using RAF-DB dataset which contains real-world expressions.

The study (Talaat, 2023) presents a real-time facial emotion detection approach for children suffering from autism spectrum disorder (ASD). ASD is a difficult-to-diagnose disorder in the early stage and facial emotions offer an alternative in this regard. Normal and ASD children are reported to show different facial emotions. The study proposes an enhanced deep learning technique based on the CNN model. Robust and improved results are reported from the proposed approach.

### 1.1 Challenges in existing approaches

Despite the improved accuracy and enhanced performance reported in the existing literature, several challenges require further efforts. To achieve accurate predictions when training neural networks, a significant amount of data is required. However, gathering datasets for subjective emotions poses a big challenge. Many databases are sourced from platforms like Amazon Mechanical Turk (AMT) or utilize hashtags of social media to label image sentiment. These methods demand substantial human effort and time, leading to increased costs. To address these issues, integrating the training dataset with synthetic images is suggested to assess whether it enhances accuracy and reduces the need for real-face images (Huang et al., 2019). Handling the variations in the human face including the color, posture, expression, etc. is challenging for a FER system. Similarly, facial muscular motions vary, as do the skin deformation from one person to another making it difficult to make a FER system capable of recognizing emotions in all scenarios. Consequently, existing FER systems suffer from low accuracy.

### 1.2 Contributions of this study

In view of the challenges pointed out earlier, this study aims to provide robust and precise results for FER. This research work creates an efficient technique for categorizing human mood from images. This study fulfills the following tasks.

1. The study proposes the use of the UNet model for image segmentation with the EfficientNetB4 transfer learning (TL) model to identify emotions including happy, sad, fear, pain, anger, and disgust.
2. Multiple experiments are performed to identify emotions. The first experiment does not involve UNet-based image segmentation. In the second experiment, UNet segmentation is performed to identify six emotions. In the third experiment, UNet segmentation is performed for binary classification involving happy and sad emotions.
3. Additionally, various ML and DL methods along with TL approaches are adopted for performance comparison. Based on the overall results with all classes (without UNet segmentation), all classes (with UNet segmentation), and binary class (with UNet segmentation), the effectiveness of the various models is assessed.

The remaining sections of the paper are arranged as follows: A summary of is given in Section 2, previous work related to human emotions for image classification. Section 3 details the dataset, including preprocessing steps and data visualization techniques employed to uncover underlying patterns within the data. This section also outlines the various algorithms utilized in the study. In Section 4, the results are discussed and analyzed. In conclusion, Section 5 summarizes the study's findings and suggests directions for further investigation.

## 2 Related works

As mentioned, major research development has been conducted on facial emotion recognition systems in the past few years. Several approaches have been developed to solve this problem. There have been approaches using features-based recognition to DL approaches (Vignesh et al., 2023; Sarvakar et al., 2023; Huang et al., 2023; Talaat, 2023). However, the CNN models are widely used for this task and reported good results concerning emotion detection from facial expressions. Qu et al. (2023) proposed a CNN-based system for FER. They used the benchmark dataset “FER 2013.” They used two different optimizers for the optimization of the CNN such as stochastic gradient descent (SGD) and Adam with different epochs. The study's results indicate that the CNN achieved a 60.20% accuracy using SGD optimizer on 00 epochs. Meena and Mohbey (2023) proposed a TL-based system for the automatic sentiment classification of images. They used the three TL models such as VGG16, Inception-v3, and XceptionNet. The authors compared the TL model's performance on three different datasets. Results of the study show that the Inception-v3 achieved the highest accuracy on the CK+ dataset which is 99.57%, VGG-19 performed well on the JAFFE dataset and attained an accuracy score of 94%, and on the FER 2013 dataset, XceptionNet achieved the accuracy score of 77.92%.

The research described in Boughida et al. (2022) introduced an innovative technique for recognizing facial expressions by utilizing evolutionary algorithms alongside Gabor filters. Facial landmarks serve to identify crucial facial areas for the extraction of Gabor features. Additionally, a genetic algorithm was employed to concurrently select optimal features and fine-tune support vector machine (SVM) hyperparameters. Regarding the JAFFE, CK, and CK+ datasets, the test results reveal the method's exceptional performance with recognition rates of 96.30%, 94.20%, and 94.26%, respectively. Gubbala et al. (2023) suggested a random forest (RF) model enhanced with AdaBoost for the classification of facial emotions from images. Their model aims to transform features from social media image posts for emotional analysis. The Adaboost-based RF model for emotion classification (ARFEC) model's core stages include class labeling, feature selection, and feature extraction. The study demonstrates that the ARFEC model achieved a peak accuracy rate of 92.57% on the CK+ dataset. Similarly, the research outlined in Oguine et al. (2022) advocated for a more efficient and accurate approach to classifying both digital and real-time facial images into one of the seven emotional categories. Enhanced training efficiency and classification accuracy are achieved through preprocessing and data augmentation methods. The proposed CNN+Haar Cascade model attained the top accuracy of 70.04% on the FER2013 dataset. Gupta and Jain (2021) developed a deep learning-based system for emotion recognition via facial expressions. By utilizing a CNN-based system inspired by the LeNet architecture, the recognition of emotions through facial features is achieved. Their study employed publicly accessible datasets featuring seven distinct categories. The proposed CNN model recorded a maximum accuracy of 60.37%.

Haider et al. (2023) proposed an innovative method for emotion classification through facial imagery. Their method features a customized ResNet18 model augmented with a triplet loss function (TLF) combined with a TL, along with an SVM model for classification purposes. This approach utilizes a facial vector and classifier to identify facial expressions by exploiting deep features from a modified ResNet trained with triplet loss. During preprocessing, facial areas are identified within the source images via Retina Face, and the features are extracted by training the ResNet18 model on cropped facial images using the triplet loss. The SVM classifier then categorizes facial expressions based on these deep features. Their results indicated that the tailored ResNet18 achieved a maximum accuracy of 99.02% on the MMI dataset. An additional study (Lucey et al., 2010) compiled the extended Cohn-Kanade dataset, which provides annotations for both emotions and action units. The dataset's performance was assessed using a combination of SVM and active appearance models (AAMs) for categorization. AAMs produce a mesh that tracks facial movements across images, yielding two feature vectors. Initially, the mesh vertices undergo translation, scaling, and rotation, followed by conversion of images to grayscale using the input photos and mesh. Through a leave-one-subject-out cross-validation process, they reported over 80% accuracy. Huang et al. (2019) proposed an advanced CNN to enhance the extraction of sentiment from images based on visual content. They significantly enhanced the training set by adding artificial face photos. The set exclusively included synthetic and genuine face images, as well as combinations of both. The result of the study shows that the AlexNet TL model achieved the highest accuracy of 87.79%. Similarly, Anilkumar et al. (2024) propose a deep CNN with hyperparameter optimization (DCNN-HPO) for correctly predicting sentiment analysis by optimizing the DCNN parameters. They used three different publicly available datasets for experiments. The VGG-16 network is used to extract features from each preprocessed image. Next, the DCNN is updated using the retrieved features, and the DCNN's weight parameters are modified via Krill Herd Optimization (KHO). Classification result shows that the proposed DCNN-HPO achieved the highest accuracy of 83.4% DCNN-HPO using the TumEmo dataset. The summary of the discussed literature is presented in Table 1.

**Table 1:** Summary of the related work.

**References**	**Classifiers**	**Dataset**	**Performance**
Qu et al. (2023)	CNN	FER 2013	60.20%
Meena and Mohbey (2023)	VGG16, Inception-v3, and XceptionNet	FER2013, JAFFE, Cohn-Kanade Dataset (CK+)	77.92% XceptionNet on FER2013, 99.57% Inception-v3 on CK+,94% VGG-19 on JAFFE
Boughida et al. (2022)	SVM kernel (Linear, RBF), Gabor filters	CK+	94.26% Gabor filter
Gubbala et al. (2023)	KNN, SVM, ARFEC	FFHQ, CK+ and FER2013	ARFEC on FFHQ = 89.5 %, CK+ = 92.5 %, FER2013= 89.5%
Oguine et al. (2022)	CNN + Haar Cascade	FER2013	70.04%
Gupta and Jain (2021)	CNN with LeNet	FER 2013	60.37%
Haider et al. (2023)	Customized ResNet18,SVM, LDA, and Softmax	JAFFE, FER2013, AFFECNET, and MMI	99.02% customized ResNet18 on MMI Dataset
Lucey et al. (2010)	AAMs, SVM	CK+	80% SVM with CAPP features
Huang et al. (2019)	AlexNet	Synthetic face dataset collected using FaceGen software	87.79%
Anilkumar et al. (2024)	ConvLSTM, MAN, AHR, DCNN-HPO	GSO-2016, MVSA-Single, TumEmo	83.4% DCNN-HPO using TumEmo

## 3 Methodology

**Figure 1:**
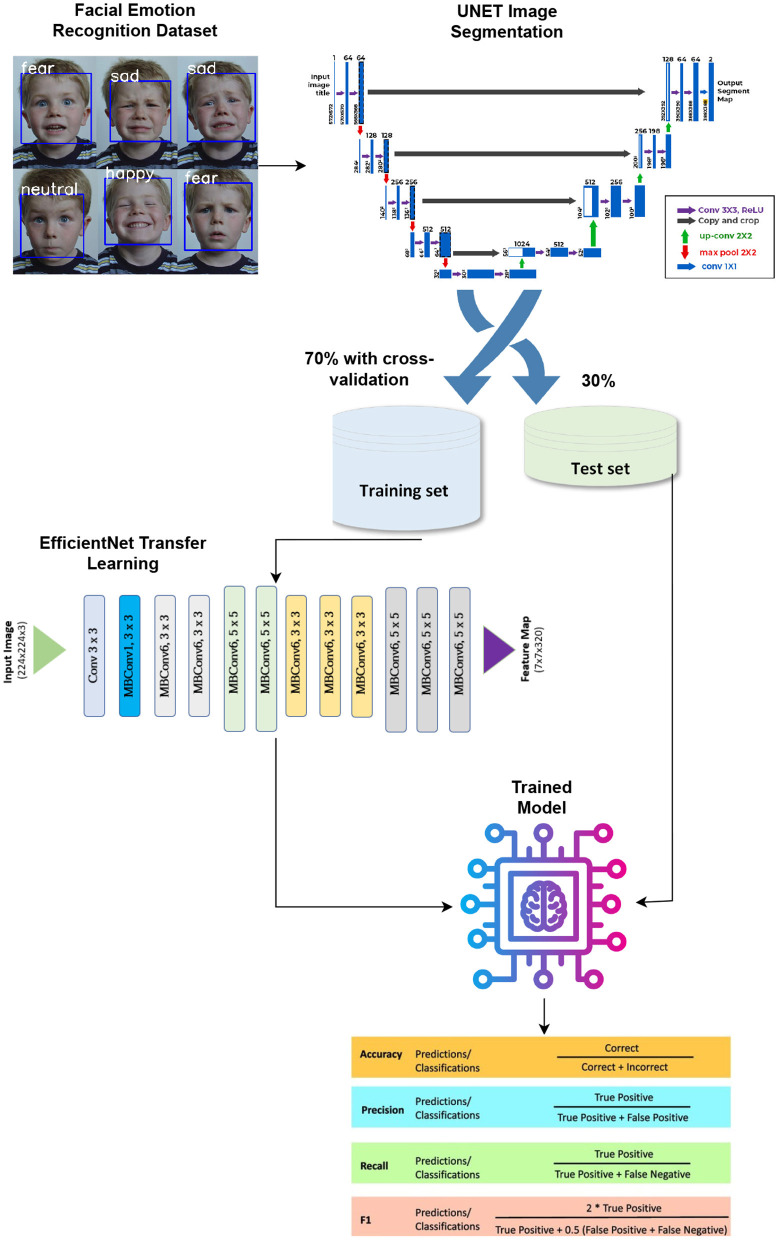
Methodological architecture of the proposed approach.

This study proposes a UNet segmentation-based TL approach employing the EfficientNetB4 model for emotion recognition. Figure 1 shows the methodological architecture of the approach.

### 3.1 Training phase

The training process for the proposed model consists of two key stages:

1. Segmentation with UNet

The UNet architecture is trained on segmented facial regions to isolate critical areas such as eyes, mouth, and facial contours. The segmentation network was optimized using the categorical cross-entropy loss function (Equation 1) with the Adam optimizer, achieving rapid convergence. The segmentation step enhances the quality of the input features for emotion classification.

2. Feature Extraction and Classification with EfficientNetB4

The segmented images are input into the EfficientNetB4 model pre-trained on the ImageNet dataset. The transfer learning approach ensures efficient feature extraction with minimal computational overhead. Fine-tuning was performed on the top layers, including a fully connected layer for emotion classification. The training parameters were optimized using the following configuration:

- Learning rate: 1 × 10^−4^
- Batch size: 32
- Epochs: 50
- Optimizer: Adam

A combination of data augmentation techniques (rotation, flipping, and scaling) was applied to improve model generalization.

### 3.2 Testing phase

The testing phase involved evaluating the model on unseen data to measure its performance on both multi-class and binary classification tasks. The metrics used for evaluation included accuracy, precision, recall, and F1-score. The testing process was carried out as follows:

- Multi-class classification: the model classified six emotions: happy, sad, fear, pain, anger, and disgust. It achieved an accuracy of 90%, outperforming prior works with 94.26%.
- Binary classification: for the classification of happy and sad emotions, the model achieved an accuracy of 96.39%, demonstrating superior performance over existing benchmarks.

The results were further validated using a five-fold cross-validation approach to ensure consistent performance across varying data splits.

### 3.3 Dataset

The Kaggle repository dataset “6 Human Emotions for image classification” contains facial images indicating people's sentiments (Mohamed, 2024). The images are in 224 × 224 dimensions, however, the position of the face varies slightly. Because of the automatic registration of the faces, every image has a face that is about in the middle and occupies a similar area. The dataset consists of six different classes such as “Happy,” “Sad,” “Fear,” “Pain,” “Anger,” and “Disgust.” There are a total of 1,200 instances that belong to six different categories. Class-wise instances of the dataset are shown in Table 2.

**Table 2:** Dataset statistics.

**Category**	**Number of instances**
Happy	230
Sad	224
Fear	163
Pain	168
Anger	214
Disgust	201

### 3.4 Dataset preprocessing

Preprocessing includes image segmentation since it allows for precise extraction of information about distinct image regions and structures. This precision in segmentation proves indispensable for a range of image classification purposes. Moreover, it paves the way for cutting-edge medical research and facilitates various clinical applications.

### 3.5 UNet for image segmentation

UNet is a convolutional neural network architecture specifically designed for image segmentation tasks. In 2015, Olaf Ronneberger, Philipp Fischer, and Thomas Brox presented it (Krithika Alias AnbuDevi and Suganthi, 2022). The name “UNet” comes from its U-shaped architecture, which is the hallmark of this network. The main objective of image segmentation is to partition an input image into multiple regions and assign each pixel to a specific class or category. The UNet architecture consists of an encoder and a decoder part. Here's a brief explanation of each of its components.

#### 3.5.1 Encoder

On the left side of the U-shaped network is the encoder, which consists of a series of convolutional and max-pooling layers. Its main function is to extract high-level features and spatial details from the input image. With increased network depth, the receptive field expands, allowing the model to recognize more intricate patterns within the image.

#### 3.5.2 Decoder

The decoder is on the right side of the U-shaped network. It consists of upsampling and convolutional layers. The decoder's role is to take the learned features from the encoder and gradually upsample the spatial resolution to produce a segmentation map. The upsampling process helps recover the spatial information lost during the downsampling in the encoder.

UNet has proven to be very effective for image-segmentation tasks because it captures both local and global contexts through the combination of encoder-decoder architecture and skip connections. It has been widely adopted and adapted for various segmentation challenges across different domains.

### 3.6 Mathematical working of UNet architecture for FER

The UNet architecture utilized in this study is composed of a contracting path (encoder) and an expansive path (decoder). Mathematically, the UNet segmentation model can be expressed as:


(1) $$\begin{array}{l} \text{Y}=f_{\text{UNet}}\left(\text{X};\theta \right), \end{array}$$

where **X** is the input image, **Y** is the segmented output, and θ represents the trainable parameters of the network. The contracting path applies convolutional layers followed by max-pooling to extract feature maps:


(2) $$\begin{array}{l} {\text{F}}_{i}=\sigma \left({\text{W}}_{i}*{\text{F}}_{i-1}+{\text{b}}_{i}\right),\;i\in \left[1,N\right], \end{array}$$

where **F**_*i*_ is the feature map at layer *i*, **W**_*i*_ and **b**_*i*_ are the weights and biases, * denotes the convolution operation, and σ is the activation function (ReLU).

The expansive path performs upsampling and concatenation of feature maps to recover spatial information:


(3) $$\begin{array}{l} {\text{F}}_{i}^{\text{up}}=\text{Up}\left({\text{F}}_{i+1}\right)⊕{\text{F}}_{i}^{\text{enc}}, \end{array}$$

where Up(·) represents the upsampling operation, and ⊕ denotes channel-wise concatenation with feature maps from the encoder (${\text{F}}_{i}^{\text{enc}}$).

### 3.7 Loss function

To optimize the UNet model for segmentation, we employed the categorical cross-entropy loss, defined as:


(4) $$\begin{array}{l} L_{\text{CCE}}=-\frac{1}{N}\overset{N}{\underset{i=1}{\sum }}\overset{C}{\underset{c=1}{\sum }}{\text{Y}}_{i,c}\log\left({\hat{\text{Y}}}_{i,c}\right), \end{array}$$

where *N* is the total number of pixels, *C* is the number of classes, **Y**_*i, c*_ is the true label, and ${\hat{\text{Y}}}_{i,c}$ is the predicted probability for class *c* at pixel *i*. This loss function ensures pixel-wise classification accuracy for multi-class segmentation.

### 3.8 ML models for emotion classification

In order to classify human emotion classification using images Classification algorithms based on ML are applied. This paper provides a brief discussion of some prominent classification methods and their theoretical foundation. The models were optimized by fine-tuning various hyperparameters for ML models.

#### 3.8.1 Random forest

For supervised learning, decision trees are improved and employed in random forests. The largest number of votes serves as the basis for this prediction criterion (Manzoor et al., 2021). Because there are improper connections between the trees in the random forest, it has a low error rate when compared to other classifiers. One way to conceptualize RF is as an ensemble model composed of several trees. The ultimate forecast of this classifier is decided by a majority vote after it generates several choice trees. Compared to decision trees, this is more efficient because decision trees collaborate and correct one another's errors. In a random forest, every tree is trained with distinct data points and has bagging. The trees are therefore unconnected to one another.

#### 3.8.2 Logistic regression

LR is a prevalent statistical technique employed for binary and multiclass classification tasks, even though its name might suggest a regression application (Rymarczyk et al., 2019). LR primarily focuses on classification. It models the connection between independent variables and the likelihood of a specific outcome occurring. By utilizing the logistic function, LR confines predictions within a range of 0–1. During the training process, LR calculates the coefficients for each independent variable through maximum likelihood estimation. These coefficients signify the influence of each variable on the probability of the outcome. LR is easily interpretable, simple to implement, and effective with data that is linearly separable. It stands as a fundamental model in the realm of machine learning.

#### 3.8.3 Extra tree classifier

The extra tree classifier (ETC) functions similarly to the RF classifier, with the exception that a random process of splitting is used in place of the top-down technique, which reduces variance by making the tree more biased (Umer et al., 2022). This is because a significant portion of the generated tree's variance is caused by the selection of the ideal cut-point. In contrast to RF bootstrapping, the ETC does not support this. For instance, the number of split points is equal to k if k attributes are chosen out of the entire N attributes in our training class. Let S represent these split sites, i.e., *S*_1_, *S*_2_, *S*_3_, *S*_*k*_. These divisions are selected at random. Every split results in a decision tree being generated. Every split yields a score representing the likelihood of choosing every class. Therefore, for class A, PA (i.e., PA1, PA2, PA3,...... Pak) provides the probability. The class with the highest probability is selected to determine the prediction, which is determined by averaging the probabilities of each class. Another name for this is majority voting. This reduction in complexity lessens the computational load on the Extra Tree Classifier and enables it to get better results in several high-dimensional complicated challenges.

#### 3.8.4 Support vector machine

SVM is a powerful supervised learning model used for challenges involving both regression and classification (Hearst et al., 1998). They work by locating the ideal hyperplane in the feature space that optimizes the distance between various classes. Support vectors or the data points nearest to the decision boundary, are what decide this hyperplane. SVMs may handle both linearly and non-linearly separable data by using kernel functions to transform the data into a higher-dimensional space. This makes it possible to create more complex decision boundaries.

### 3.9 DL models for emotion classification

Artificial intelligence methods that imitate human knowledge acquisition and DL are connected to ML approaches. DL is an essential part of data science, which encompasses statistics and predictive modeling. One kind of deep neural network utilized in deep understanding is the CNN, which analyzes visual data. CNN is a DL method that employs weights to recognize various objects in an input image so it can make distinctions between them. CNN is used to categorize and identify photos due to its high degree of accuracy.

DL architectures ResNet, MobileNet, VGG19, EfficientNetB4, and InceptionV3- are employed to categorize the data. These models are trained by TL. A total of 50 training epochs have been used to train each model. Below is a thorough explanation.

#### 3.9.1 Convolutional neural network

CNNs are based on the visual system of the human brain. CNNs therefore aim to make computers able to see the world as humans do (Lu et al., 2021; Ding et al., 2023). CNNs can thus be applied to NLP, image classification, and diagnosis. CNN is a subset of DNN that has nonlinear activation layers, max pooling, and convolutional layers. The convolutional layer, which is thought to be responsible for the “convolution” operation that gives CNN its name, a CNN's primary layer. Layer inputs are subjected to the application of convolutional layer kernels. A feature map is created by convolving each of the convolutional layers' outputs. Since the input images in this study are inherently nonlinear, the ReLU activation function together with maxpooling layers, helps to augment the non-linearity in the image. Therefore, in the current scenario, CNN with ReLU is straightforward and faster. The ReLU can be defined as follows because it is 0 for all negative inputs:


(5) $$\begin{array}{l} z=max\left(0,i\right) \end{array}$$

where *z* shows the output and *max* calculates the maximum value from 0 and input value *i*. In this case, the function suggests that the positive value stays constant and the output *z* is zero for all negative values.

#### 3.9.2 VGG19

The 19-layer VGG19 model is a deep CNN. For tasks involving picture classification, it is trained using the ImageNet dataset (Rajinikanth et al., 2020; Cao et al., 2024). A 2 × 2 max pooling layer and a ReLU activation function come after each repeating 3 × 3 convolutional layer in the architecture. VGG19 is frequently utilized in computer vision research due to its high accuracy on a variety of picture classification benchmarks. Nevertheless, because of its many characteristics, it is computationally costly and challenging to implement on devices with limited resources.

#### 3.9.3 ResNET

Residual Network (ResNet) is a type of CNN that is commonly used for TL, especially in the context of DL for image processing tasks such as image recognition and classification (Yaqoob et al., 2021). ResNet revolutionized DL by enabling the training of extremely deep neural networks with 152 layers or more. Before the introduction of ResNet, such deep networks were hard to train due to the vanishing gradient problem, where the gradient signal gets smaller and smaller as it backpropagates through each layer, eventually becoming too tiny to make any significant changes in the weights in the lower layers. ResNet addresses this by using skip connections, or shortcuts to jump over some layers. The outputs of these connections, which carry out identity mapping, are added to the stacked layer outputs, effectively allowing the training signal to be directly propagated back through the network. This design makes it possible to train very deep networks, and thus ResNet models can learn richer and more complex feature representations. A ResNet model that has been pre-trained on a large and general dataset like ImageNet is often used as a starting point for a new task. Because the initial layers of a CNN tend to learn features that are generally useful for analyzing images, such as edges and textures, they can be effectively applied to new tasks with little alteration. The later layers of the network, which learn more specific patterns, may be fine-tuned with a smaller dataset specific to the new task, ensuring adaptability and relevance.

#### 3.9.4 EfficientNetB4

CNN architecture EfficientNetB4 was created to minimize the amount of computing power needed for training and deployment while achieving good accuracy on image recognition (Park et al., 2022). After being trained on the ImageNet dataset, EfficientNetB4 performs well on several image recognition benchmarks. Multiple pooling layers with activation functions are included in the model design. With fewer parameters and increased training efficiency, it additionally combines depthwise and pointwise convolutions. Because of its strong transferability to different tasks and datasets, EfficientNetB4 is a valuable tool for TL. However, for effective deployment and training, specific hardware could be needed.

#### 3.9.5 MobileNet

MobileNet uses depth-wise separable convolutions to significantly reduce the number of parameters compared to standard convolutions of the same depth (Srinivasu et al., 2021; Zhu, 2024). Consequently, lightweight deep neural networks are generated. Mobile networks are built using depth-wise separable convolution layers. A pointwise convolution layer plus a depth-wise convolution layer make up each depth-wise detachable convolution layer. MobileNet has 28 layers. Through the manipulation of the width multiplier hyperparameter, a conventional MobileNet can contain as few as 4.2 million parameters. The input image measures 224 by 224 pixels.

#### 3.9.6 InceptionV3

The Inception-V3 model optimizes the network using multiple methods for increased model adaptability (Mujahid et al., 2022). As compared to the V1 and V2 inception models, V3 has a larger network. The DNN model Inception-V3 is trained directly on lesser parameters.

### 3.10 Evaluation parameters

The evaluation phase, which includes evaluating learning models' performance, is critical to performance analysis. Standard assessment measures such as F1 score, recall, accuracy, and precision are used to evaluate FacialNet model performance (Umer et al., 2022).

Accuracy is the most commonly used performance metric. It is just the ratio of observations that were successfully predicted to all observations. It works well with problems involving binary and multi-class classification.


(6) $$\begin{array}{l} Accuracy=\frac{\left(Number\;of\;correct\;pridictions\right)}{\left(Total\;number\;of\;predictions\right)} \end{array}$$

(7) $$\begin{array}{l} Accuracy=\frac{TP+TN}{TP+TN+FP+FN} \end{array}$$

where *TP* and *TN* show true positives and true negatives, respectively while *FP* and *FN* indicate false positives and false negatives, respectively.

Precision is known as positive predictive value, and it is crucial in situations where the expenses associated with false positives are substantial.


(8) $$\begin{array}{l} Precision=\frac{TP}{TP+FP} \end{array}$$

The percentage of accurately predicted positive observations is known as a recall to all actual class yes observations. When the expense of false negatives is substantial, it matters.


(9) $$\begin{array}{l} Recall=\frac{TP}{TP+FN} \end{array}$$

The F1 Score is a harmonic mean of precision and recall.


(10) $$\begin{array}{l} F1-score=2\times \frac{Precision\times Recall}{Precision+Recall} \end{array}$$

## 4 Experiments and results

For human emotions classification extensive experiments are carried out. ML and DL models are applied using the six different classes without UNet segmentation, six different classes with UNet segmentation, as well as, the two classes with UNet segmentation. Results and detailed discussion are analyzed in this section.

### 4.1 Experimental setup and system specifications

The Python 3.9 programming environment is used to conduct the research. The study's experimental setting includes the computer language Python 3.8, (Scikit learn version Version 1.5.0 and TensorFlow version r2.15), RAM capacity available (8GB DDR4), operating system type (64-bit Windows 11), CPU specifications are Intel Core i7 with a processor frequency at about 2.8 GHz which belongs to the 7th generation and an Nvidia GTX1060 GPU. This information is relevant for comprehending the technical characteristics of the research setting and the computational resources employed in this study. The ML classifiers' performances were evaluated using various performance evaluation metrics.

### 4.2 Results of multiclass without UNet segmentation

The first stage of the experiments involved six classes including happy, sad, fear, pain, anger, and disgust classes without UNet segmentation and ML and TL models. Table 3 provides a summary of the models' performance.

**Table 3:** Without UNet segmentation six classes (happy, sad, fear, pain, anger and disgust) classification.

**Model**	**Accuracy**	**Precision**	**Recall**	**F1-score**
LR	72.40	73.24	73.39	73.31
RF	70.22	71.37	72.42	72.40
ETC	70.42	71.44	72.14	71.89
SVM	72.49	71.93	70.64	71.09
CNN	74.57	75.74	75.89	75.81
VGG19	69.89	72.17	73.76	72.99
ResNET	73.24	72.52	73.34	72.86
EfficientNetB4	81.56	80.96	81.23	81.19
MobileNet	80.15	81.67	80.54	81.09
InceptionV3	79.95	80.25	80.25	80.25

The study showed that TL models EfficientNetB4, MobileNet, and InceptionV3 had the highest accuracy rates. EfficientNetB4 outperformed all other models in accuracy. Overall, the performance of all models using six emotion classes without UNet segmentation data was unsatisfactory.

### 4.3 Results of models on multiclass with UNet segmentation

Regarding the subsequent series of tests, the six-class classification with UNet segmentation in the dataset is used. Many ML and DL models were trained and tested using the resultant dataset. Table 4 illustrates the performance of various models with the UNet segmentation. It shows that ML models have shown the lowest results as compared to the DL and TL models respectively.

**Table 4:** With UNet segmentation six classes (happy, sad, fear, pain, anger and disgust) classification.

**Models**	**Accuracy**	**Precision**	**Recall**	**F1-score**
LR	79.56	80.37	80.93	80.77
RF	78.16	79.73	80.24	79.97
ETC	80.46	81.17	81.14	81.15
SVM	80.94	81.48	80.17	81.11
CNN	84.74	85.24	85.30	85.27
VGG19	79.25	79.71	80.24	80.15
ResNET	83.36	85.17	85.67	85.42
EfficientNetB4	90.11	90.34	91.27	91.05
MobileNet	88.53	89.86	89.94	89.90
InceptionV3	89.66	90.11	90.07	90.10

Table 4 presents the performance metrics of various models, ranging from traditional machine learning models like LR and RF to advanced deep learning models such as CNN, VGG19, ResNet, and EfficientNetB4 for emotion recognition tasks. Traditional models like LR and RF achieved relatively low accuracies of 79.56 and 78.16%, respectively, as these models have limited capacity to capture complex patterns in high-dimensional image data. The ETC performed slightly better with an accuracy of 81.17%, and SVM further improved with 80.94%, which can be attributed to its strong ability to handle high-dimensional spaces.

In contrast, DL models showed a marked improvement, with CNN achieving 84.74% accuracy, demonstrating its effectiveness in capturing spatial hierarchies in images. ResNet and InceptionV3 continued this trend, with ResNet scoring 85.67% and InceptionV3 reaching 90.07%, thanks to their deeper architectures and additional design elements like residual connections and inception modules. EfficientNetB4, however, stood out with the highest accuracy of 91.27%, due to its compound scaling approach that optimally balances network depth, width, and resolution. MobileNet, while designed for mobile devices, still performed well with 89.94% accuracy, though it trailed behind EfficientNet. VGG19, on the other hand, performed similarly to traditional models with an accuracy of 79.71%, suggesting that depth alone is not enough for superior performance, and additional architectural innovations are essential. Overall, deep learning models, especially EfficientNetB4, significantly outperformed traditional methods in the emotion recognition task.

The integration of UNet segmentation in the proposed model significantly enhances the overall performance by providing a refined preprocessing step that isolates relevant facial features, enabling the classifier to focus on key regions of interest related to emotions. The UNet architecture's encoder-decoder design excels in capturing detailed spatial features, enabling precise segmentation of facial regions such as eyes, mouth, and forehead, which are essential for emotion detection. This enhances the input quality for subsequent models, like EfficientNet, leading to improved accuracy and generalization. However, segmentation does come with its challenges. The final classification accuracy is significantly affected by segmentation quality; poor outcomes can cause errors or omit critical emotional cues, especially in situations with occlusions, varying lighting, or unusual facial expressions. Additionally, segmentation increases computational demands, potentially impacting real-time performance in practical applications. Yet, when used with advanced models like EfficientNet, the precise feature extraction benefits generally surpass the computational costs, making it advantageous for detailed facial analysis tasks like Facial Emotion Recognition (FER). The evaluation showed that the transfer learning models EfficientNetB4, MobileNet, and InceptionV3 classifiers attained accuracy rates of 90.11%, 88.53%, and 89.66%, respectively. The results demonstrated significant enhancements in learning model performance using UNet segmentation across the six classes, with noticeable improvements in machine learning models on the segmented dataset compared to data without UNet segmentation. The EfficientNetB4 model achieved the highest accuracy. Furthermore, its precision, recall, and F1 scores were 90.34%, 91.27%, and 91.05% respectively. The RF linear model showed the lowest accuracy at 78.16%. The development of the FacialNet model using UNet coupled with EfficientNetB4 leverages the distinct advantages of each framework for emotion recognition and mental health evaluation. UNet is adept at segmenting images to pinpoint crucial facial features indicative of emotional states. Its segmentation accuracy allows for a focused examination of important facial areas. Conversely, EfficientNetB4, with its effective feature extraction and pre-trained weights, captures intricate patterns in high-dimensional facial datasets efficiently. This hybrid model, fusing UNet's segmentation skills with EfficientNetB4's feature extraction capabilities, achieves a commendable balance between accuracy and computational cost-ideal for emotion recognition tasks in mental health contexts.

### 4.4 Binary classification results with UNet segmentation

The experiments involved binary classification on datasets segmented with UNet. Given the dataset contains six classes, the outcomes for multi-class classification were unsatisfactory. Consequently, So, in this set of experiments we treated two classes such as happy and sad (Sad, Fear, Pain, Anger, and Disgust) classes, and performed the binary classification on this type of dataset with UNet segmentation results of the learning models are shown in Table 5.

**Table 5:** Binary class (“positive,” and “negative”) classification with UNet segmentation.

**Models**	**Accuracy**	**Precision**	**Recall**	**F1 score**
RF	87.55	88.64	88.19	88.42
LR	89.24	90.54	90.48	90.52
ETC	90.22	91.29	91.41	91.34
SVM	90.57	91.14	90.79	91.04
CNN	92.24	93.25	93.90	93.52
VGG19	89.64	90.56	90.42	90.49
ResNET	94.44	95.42	94.19	94.88
EfficientNetB4	96.39	96.88	97.39	97.27
MobileNet	94.28	93.10	94.04	93.97
InceptionV3	95.24	94.87	95.17	95.11

Results of the experiments show that the proposed EfficientNetB4 with the UNet segmentation features outperformed the other learning models and achieved an accuracy of 96.39%. Followed by InceptionV3 achieved an accuracy of 95.24%. The proposed TL model EfficientNetB4 achieved the highest value for the other evaluation parameters, 96.88% precision, 97.39% recall, and an F1 score of 97.27%. In this part of the experiment, a notable improvement in the performance of ResNet is noted. In this part of the experiment ML model, RF is the least performer and achieved an accuracy of 87.55%. Overall, there is a significant improvement in the performance of the learning classifiers used for the emotions classification is noted.

### 4.5 Results for statistical significance test

To further evaluate the differences in performance among the models, a paired *t*-test was conducted between EfficientNetB4, MobileNet, and InceptionV3. The results demonstrated in Table 6 show that the difference in performance is statistically significant. EfficientNetB4 significantly outperforms both MobileNet and InceptionV3 across accuracy, precision, recall, and F1-score. The *p*-values for both comparisons are below 0.05, confirming that EfficientNetB4 provides a substantial performance improvement over the other models.

**Table 6:** Paired *t*-test results between the models.

**Model Comparison**	***t*-statistic**	***p*-value**
EfficientNetB4 vs. MobileNet	8.75	0.0031
EfficientNetB4 vs. InceptionV3	7.57	0.0048

### 4.6 Cross-validation technique results

One method for assessing how well ML algorithms work is cross-validation. Although there are other cross-validation techniques, *k*-fold cross-validation is preferred because it is well-liked, simple to comprehend, and typically produces less bias than the other techniques.

The choice of *k* = 5 strikes a balance between computational efficiency and reliable estimation. Larger *k*-values (such as 10) would provide even more precise performance estimates but at the cost of increased training time. On the other hand, smaller *k*-values (such as 2 or 3) might not provide enough diversity in training and validation sets, leading to less reliable generalization performance. By choosing *k* = 5, the method ensures a comprehensive evaluation while keeping computational demands manageable, enhancing the robustness and reliability of the reported results.

The data set is split into *k* equal-sized portions for *k*-fold cross-validation. The first *k* groups are used to train the classifiers, while the remaining portion is utilized to evaluate outperformance at each stage. There are *k* repetitions of the validation process. Based on *k* outcomes, the classifier performance is calculated. Various values of *k* are chosen for cross-validation. Since *k*=5 performs well, we employed it in the experiments. 90% of the data in the five-fold cross-validation procedure were used for training, while 10% were used for testing. All occurrences in the training and test groups were randomly distributed throughout the whole dataset before selecting, training, and testing fresh sets for the following cycle. This process was performed five times for each fold of the process. Finally, averages of all performance metrics are calculated after the five-fold process. The suggested TL model EfficientNetB4 yields a mean accuracy score of 96.68, and average scores of 97.45, 97.52, and 97.50 for precision, recall, and F1, in that order, according to the cross-validation results displayed in Table 7.

**Table 7:** UNet segmentation cross-validation results.

**Models**	**Accuracy**	**Precision**	**Recall**	**F1-score**
First*-fold*	96.33	96.54	97.34	97.62
Second*-fold*	97.84	97.67	98.58	98.59
Third*-fold*	97.77	97.19	97.49	97.49
Fourth*-fold*	96.68	97.49	97.59	97.38
Fifth*-fold*	96.71	97.88	97.89	97.49
**Average**	**96.68**	**97.45**	**97.52**	**97.50**

### 4.7 Performance comparison with previous approaches

In Table 8, we performed a comparison with other studies that have previously worked on facial emotion classification to highlight the importance of the suggested method. The accuracy attained in those earlier tests showed a significant gap, indicating that there is a great deal of space for accuracy improvement. The majority of earlier research leveraged TL approaches and concentrated on using the facial expression image dataset directly. This study, on the other hand, made use of UNet segmentation characteristics that were taken from an image dataset. The suggested EfficientNetB4 model produced highly significant results by utilizing this feature set, as seen in the Table 8. This indicates that the suggested method is effective in outperforming the results of earlier research and points to the possibility of more developments in this area.

**Table 8:** Performance comparison with previous approaches.

**References**	**Classifiers**	**Accuracy**	**Limitations**
Qu et al. (2023)	CNN	60.20%	No prepossessing, No multi-class classification, No segmentation, No cross-validation
Boughida et al. (2022)	Gabor filter	94.26%	No prepossessing, No multi-class classification, No segmentation, No cross-validation
Gubbala et al. (2023)	ARFEC	92.5 %	No prepossessing, No segmentation, No cross-validation
Oguine et al. (2022)	CNN + haar cascade	70.04%	No prepossessing, No multi-class classification, No segmentation, No cross-validation
Gupta and Jain (2021)	CNN with LeNet	60.37%	No prepossessing, No segmentation, No cross-validation
Lucey et al. (2010)	SVM with CAPP features	80.00%	No prepossessing, No segmentation, No cross-validation
Huang et al. (2019)	AlexNet	87.79%	No prepossessing, No segmentation, No cross-validation
Anilkumar et al. (2024)	DCNN-HPO	83.40%	No multi-class classification, No cross-validation
**Proposed**	**EfficientNetB4**	**96.39%**	Computational complexity

Bold values indicating the results of the proposed approach which are better than all previously published research works.

Facial emotion recognition using CNN in Qu et al. (2023) utilizes a traditional CNN architecture, achieving moderate performance. In contrast, FacialNet's combination of UNet segmentation and EfficientNetB4 enhances feature extraction accuracy, leading to superior results, particularly for complex emotion datasets. Similarly, Meena and Mohbey (2023) explore transfer learning models for sentiment analysis, but their lack of specialized segmentation, like UNet, limits their performance. FacialNet's segmentation provides more refined facial feature representation, achieving 96% accuracy for binary classification. The study by Boughida et al. (2022) uses Gabor filters and a genetic algorithm for feature selection, which, although effective, falls short in real-time applications where FacialNet's deep learning approach offers faster and more accurate predictions. AdaBoost-based RF models in Gubbala et al. (2023) exhibit inferior performance when compared to the cutting-edge deep learning architecture of FacialNet, which employs the EfficientNetB4 backbone combined with UNet segmentation to achieve notably superior outcomes. In addition, although Oguine et al. (2022) presents a hybrid FER model, it lacks the use of advanced segmentation strategies, resulting in inferior emotion classification accuracy compared to FacialNet, which outperforms due to its precise segmentation and broad emotion classification capabilities.

### 4.8 Practical implications of proposed approach

This research significantly enhances the field of facial emotion recognition, with notable applications in mental health assessments and emotion-aware technologies. The integration of UNet segmentation with EfficientNet boosts accuracy by capturing detailed facial nuances, rendering the model highly suitable for practical applications such as telemedicine and adaptive educational settings. Future advancements could involve testing with varied datasets, inclusion of multi-modal data, and real-time optimization for use on mobile or embedded platforms. Additional future research directions might focus on further optimizing the model design, investigating supplementary features, and assessing larger, more varied datasets to improve the model's resilience and ability to generalize effectively.

## 5 Conclusion

This research proposes FacialNet for human FER using UNet image segmentation in conjunction with TL utilizing the EfficientNetB4 model. The proposed model has demonstrated impressive performance, achieving an accuracy score of 90% for six emotion classes including happy, sad, fear, pain, anger, and disgust, and 96% for binary classification including positive and negative classes. Through extensive experimentation and comparison with other ML and DL models, as well as state-of-the-art previous research works, we have validated the effectiveness and superiority of our proposed approach. Furthermore, the robustness and generalization capability of the proposed model have been thoroughly evaluated using a five-fold cross-validation technique. This validation methodology ensures the reliability and consistency of our results across different data splits, highlighting the significance and reliability of the proposed approach. The findings indicate that leveraging UNet image segmentation and EfficientNetB4 TL yields promising outcomes in the domain of FER, paving the way for the development of more accurate and efficient emotion recognition systems in various real-world applications.

## Data Availability

The original contributions presented in the study are included in the article/supplementary material, further inquiries can be directed to the corresponding author.
